# Supplementary material for: Modeling the cell biology of PEX11β deficiency during human neurogenesis
Source: bioRxiv. 2026 Feb 16:2026.02.13.705842. Preprint. [Version 1] doi: 10.64898/2026.02.13.705842 (PMC12934825; doi:10.64898/2026.02.13.705842)

Supplemental Figure Legends

Supplementary Figure 1. Validation of PEX11β KO iPSCs. (A) qRT-PCR analysis of the PEX11 isoforms, PEX11β, PEX11α, and PEX11γ, in control and PEX11β KO iPSCs relative to control and normalized to two housekeeping genes (*GPI* and *GAPDH)*. Each dot on the graph represents a biological replicate; different colors represent different clones (n=3). Analyzed using Welch's t-test; error bars represent mean ± SEM. **(B)** Whole genome sequencing results confirming exonic, frameshift deletions in two PEX11β KO clones, resulting in early stop codons (denoted by fs*30 or fs*31). **(C)** Mass spectrometry analysis showing 40% sequence coverage of the PEX11β sequence in control iPSCs, with no detection of peptides matching PEX11β in PEX11β KO iPSCs. (**D)** Karyotyping indicates that control and PEX11β KO iPSC clones have a normal number and structure of chromosomes.

Supplementary Figure 2. PEX11β KO iPSCs maintain stem cell identity. (A) PluriTest score showing that both control and PEX11β KO iPSCs share a similar pluripotency signature compared to a non-iPSC control. Pluripotency score (y-axis) indicates how strongly the model-based pluripotency signature is expressed in the analyzed samples. Novelty score (x-axis) shows how well the transcriptional profile of the cells of interest matches the expected transcriptional profile. **(B)** qRT-PCR analysis of stem cell identity markers *OCT4*, *NANOG*, and *SOX2* in control and PEX11β KO iPSCs relative to control and normalized to two housekeeping genes (*GPI* and *GAPDH)*. Each dot on the graph represents a biological replicate; different colors represent different clones (n=3). Analyzed using Welch's t-test; error bars represent mean ± SEM. **(C)** Representative maximum intensity projections of peroxisomal morphology (PEX14) and mitochondrial morphology in control and PEX11β KO iPSCs. Scale bars = 10µm (SoRa).

Supplementary Figure 3. PEX11β KO iPSCs differentiate into the three germ layers. (A) Representative maximum intensity projections of control and PEX11β KO iPSCs

differentiated into endoderm (SOX17, FOXA2). Scale bars = 100μm (SDC). **(B)** Representative maximum intensity projections of control and PEX11β KO iPSCs differentiated into mesoderm (BRACHYURY, NCAM). Scale bars = 100μm (SDC). **(C)** Representative maximum intensity projections of control and PEX11β KO iPSCs differentiated into ectoderm (PAX6, NESTIN). Scale bars = 100μm (SDC).

Supplementary Figure 4. Peroxisome number is reduced in PEX11β-deficient NPCs but not iPSCs. (A) Representative maximum intensity projections of peroxisomal morphology (PEX14) in control and PEX11β KO iPSCs. Scale bars = 10μm. Zoom scale bars = 2.5μm (SoRa). **(B)** Quantification of peroxisomal count in control and PEX11β KO iPSCs normalized to DAPI. Each dot represents the average per field of view (7 per n), each triangle represents a biological replicate, and different colors represent different clones (n=3). Analyzed using Welch's t-test; error bars represent mean ± SEM. **(C)** Representative maximum intensity projections of peroxisomal morphology (PEX14) in control and PEX11β KO NPCs at day 8 of differentiation. Scale bars = 10μm. Zoom scale bars = 2.5μm (SoRa). **(D)** Quantification of peroxisomal count in control and PEX11β KO NPCs at day 8 of differentiation, normalized to DAPI. Each dot represents the average per field of view (7 per n), each triangle represents a biological replicate, and different colors represent different clones (n=3). Analyzed using Welch's t-test; error bars represent mean ± SEM.

Supplementary Figure 5. Peroxisomal fission is reduced in PEX11β KO NPCs. (A) Representative still images from live 3D time-lapse imaging of peroxisomes in control NPCs at day 4 of differentiation using Peroxisome Cell Light (n=3, 5 cells per replicate). Cells were imaged at 5-second intervals for 15 minutes (final minute excluded from analysis due to photobleaching). Magenta arrows show points of observed peroxisomal fission. PEL overlay is rendered on frame 1 to indicate which structure undergoes fission in frame 2 and is maintained for an additional frame. Scale bars = 10μm (SDC). **(B)** Still images from live imaging of peroxisomes in PEX11β KO NPCs using Peroxisome Cell Light (n=3, 5 cells per

replicate). Cells were imaged at 5-second intervals for 15 minutes (final minute excluded from analysis due to photobleaching). Magenta arrows show points of observed peroxisomal fission. PEL overlay is rendered on frame 1 to indicate which structure undergoes fission in frame 2, with fission maintained for the subsequent frame. Scale bars = 10µm (SDC). **(C)** Quantification of peroxisome fission events in the first 100 frames of peroxisome live imaging in control and PEX11β KO NPCs. Each dot on the graph represents a biological replicate. Analyzed using paired t-test; error bars represent mean ± SEM.

Supplementary Figure 6. Colocalization of MFF, FIS1, and DRP1 with PMP70 in NPCs. A) Representative maximum intensity projections of MFF, mitochondria (MitoTracker), and peroxisomes (PMP70) in control and PEX11β KO NPCs at day 8 of differentiation. Scale bars = 10µm (SoRa). **(B)** Representative maximum intensity projections of FIS1, mitochondria (MitoTracker), and peroxisomes (PMP70) (SoRa) in control and PEX11β KO NPCs at day 8 of differentiation. Scale bars = 10µm. **(C)** Representative maximum intensity projections of DRP1, mitochondria (MitoTracker), and peroxisomes (PMP70) in control and PEX11β KO NPCs at day 8 of differentiation. Scale bars = 10µm (SoRa).

Supplementary Figure 7. Control and PEX11β KO neural rosettes. A-C) Representative maximum intensity projections of PAX6 (neural progenitors) and ZO-1 (lumen) in control and PEX11β KO neural rosettes at day 8 of differentiation. Scale bars = 100µm (SDC).

Video S1, related to Figure 5. Peroxisomal fission events in control NPCs. Peroxisomal event localizer detection of peroxisomal fission events (red) in control NPCs. 3D time-lapse acquisition every 5 seconds for 15 minutes (final minute excluded due to photobleaching). Scale bars = 10µm (SDC).

Video S2, related to Figure 5. Peroxisomal fission events in PEX11β KO NPCs. Peroxisomal event localizer detection of peroxisomal fission events (red) in PEX11β KO

NPCs. 3D time-lapse acquisition every 5 seconds for 15 minutes (final minute excluded due to photobleaching). Scale bars = 10μm (SDC).

Supplementary Figure 1

A

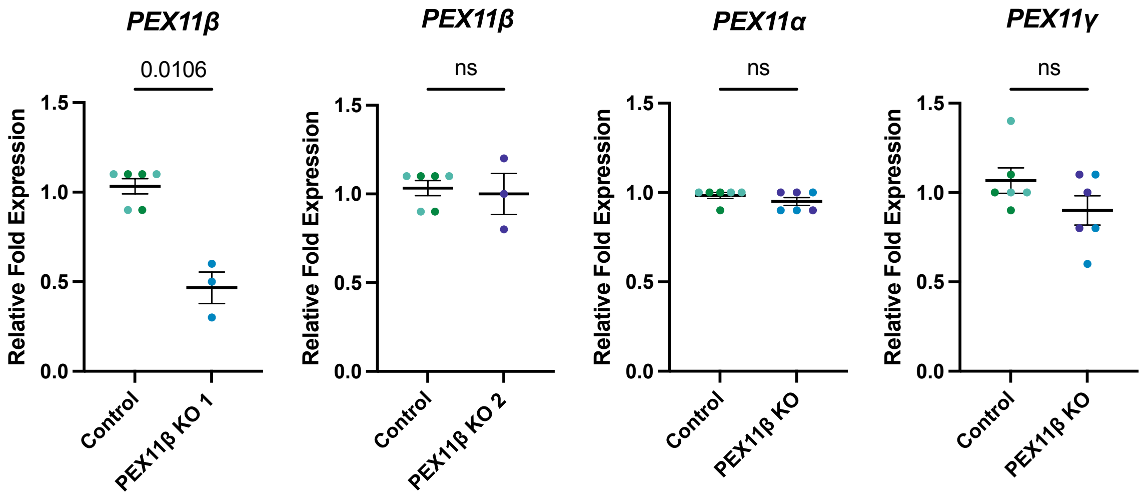

B

Key
PEX11β KO 1
PEX11β KO 2

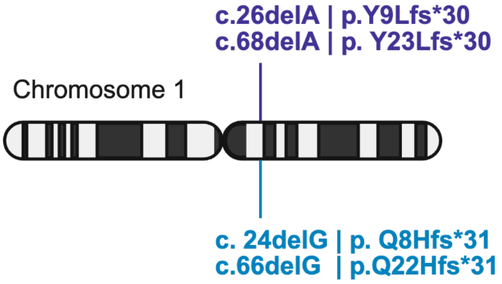

	Priority	Function	ExonicFunc	Exon Position
PEX11β KO 1	High	Exonic	Frameshift deletion	Exon 2
PEX11β KO 2	High	Exonic	Frameshift deletion	Exon 2

C

Control
PEX11β KO 1
PEX11β KO 2

PEX11B – Human

MDAWVR**FSAQSQAR**ERLCRAAQYACSLLGHALQR**HGASPELQ**KQIRQLESHLSLGR
KLLR**LGNSADALESAK**R**AVHLSDVVLR**FCITVSHLNRALYFACDNVLWAGKSGLAPR
VDQEKWAQRSFRYYLFSLIMNLSRDAYEIR**LLMEQESSACSR**RLKGSGGGVPGGSE
TGGLGGPGTPGGGLPQLALKLR**LQVLLLAR**VLR**GHPPLLLDVVR****NACDLFIPLDK**LG
LWRCGPGIVGLCGLVSSILSILTLIYPWLRLKP

D

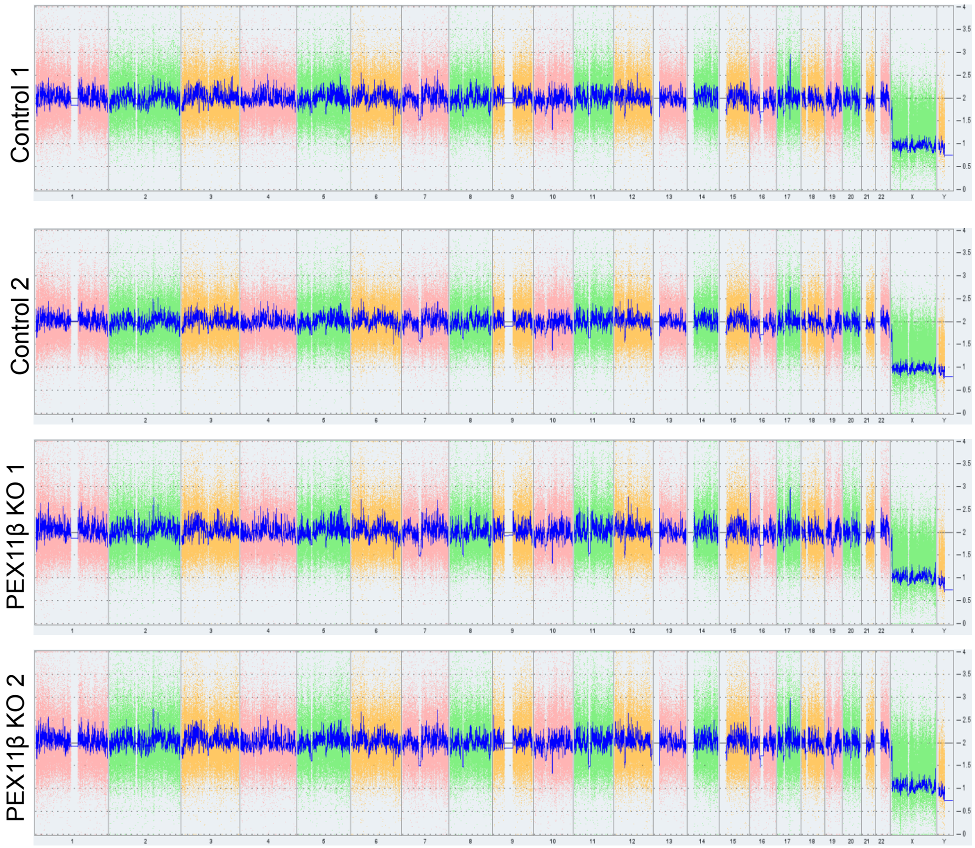

Supplementary Figure 2

A

Sample	PluriTest Result	Pluripotency Score	Novelty Score
Control 1	Pass	40.2785	1.595706
Control 2	Pass	41.47454	1.577171
PEX11β KO 1	Pass	38.52407	1.620435
PEX11β KO 2	Pass	39.58702	1.570617
iPSC Control	Pass	37.90687	1.229926
Non-iPSC Control	Fail	-45.36237	2.718222

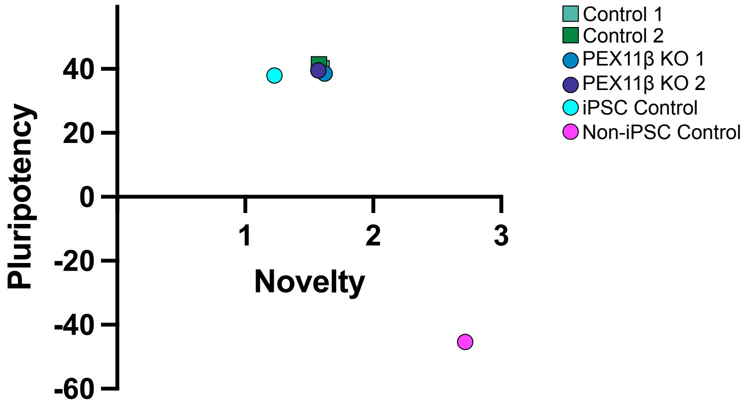

B

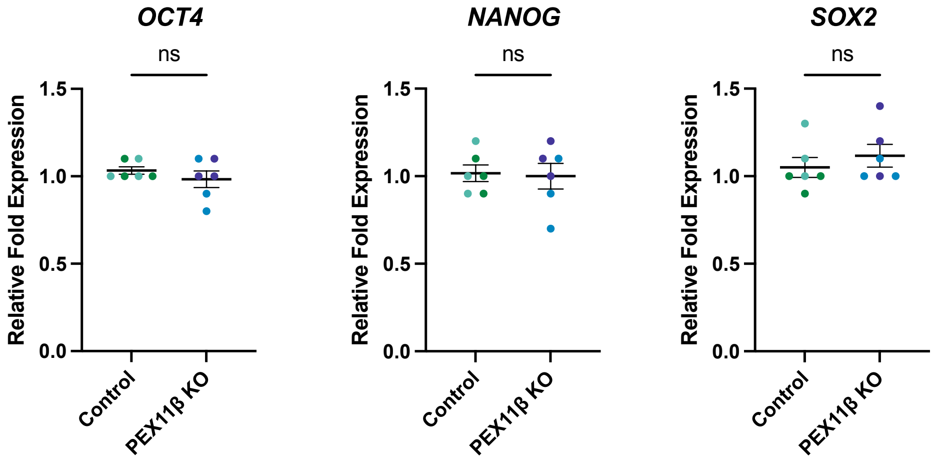

C

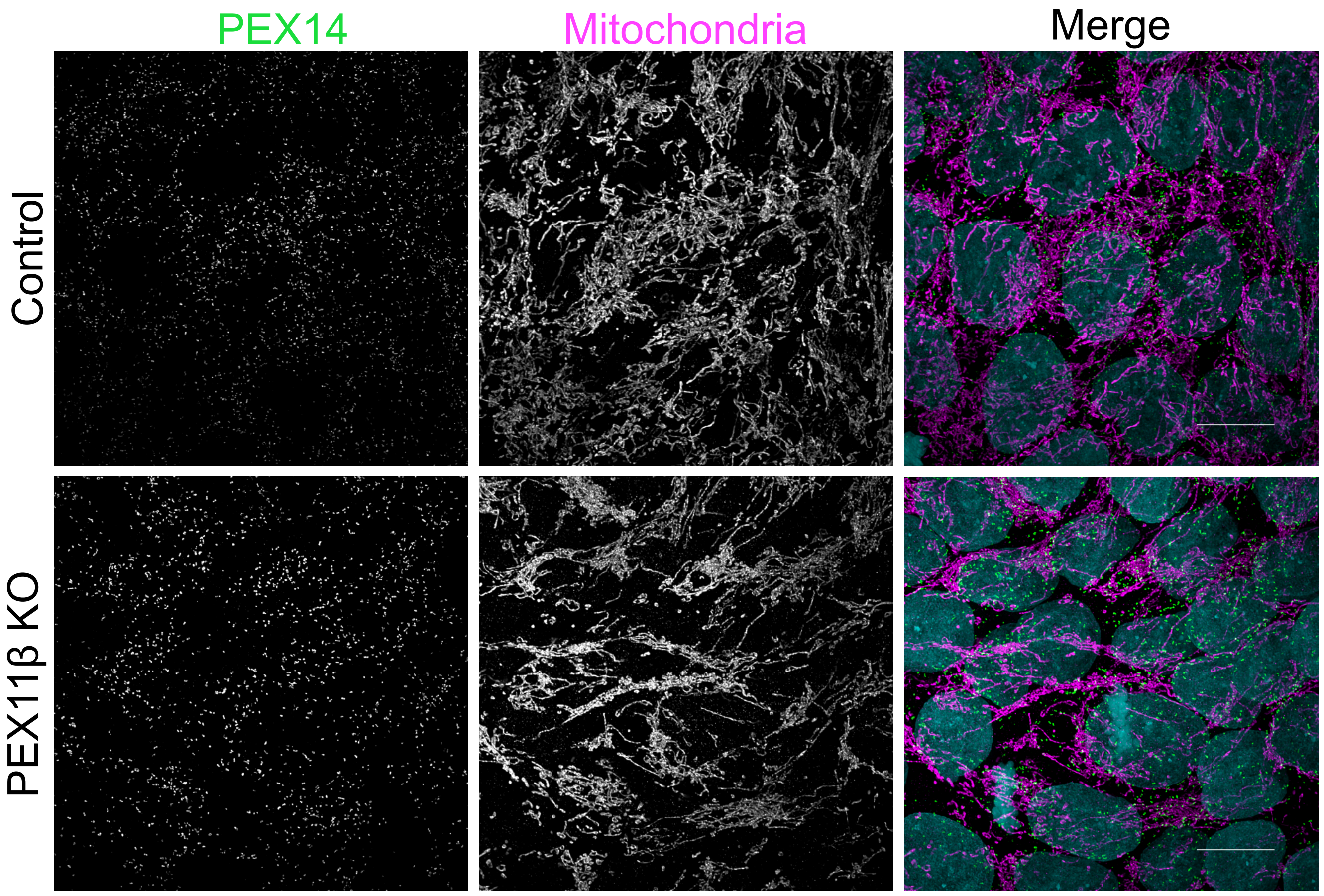

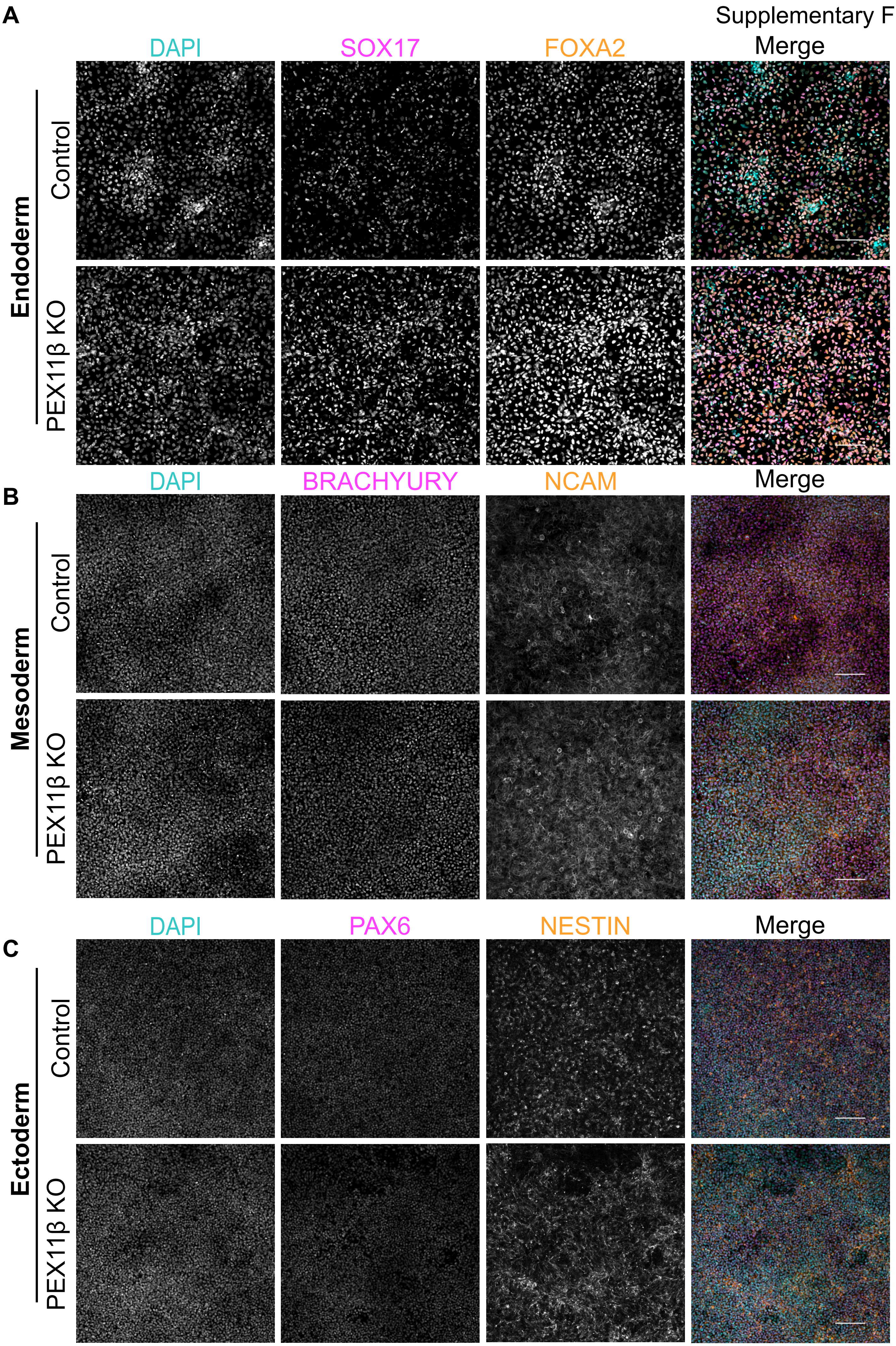
A
DAPI
SOX17
FOXA2
Merge
Endoderm
Control
PEX11β KO
B
DAPI
BRACHYURY
NCAM
Merge
Mesoderm
Control
PEX11β KO
C
DAPI
PAX6
NESTIN
Merge
Ectoderm
Control
PEX11β KO

Supplementary Figure 4

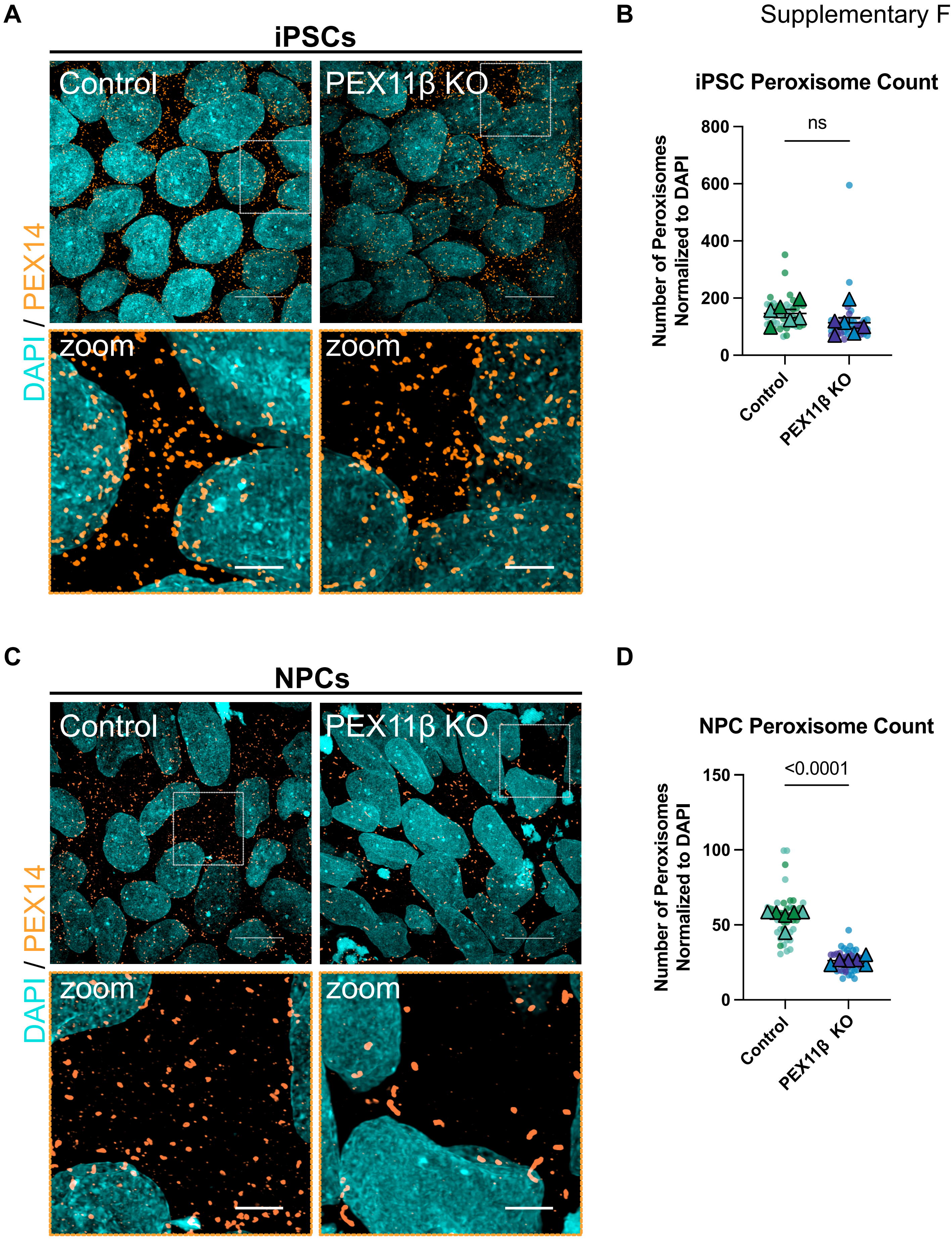

Supplementary Figure 5

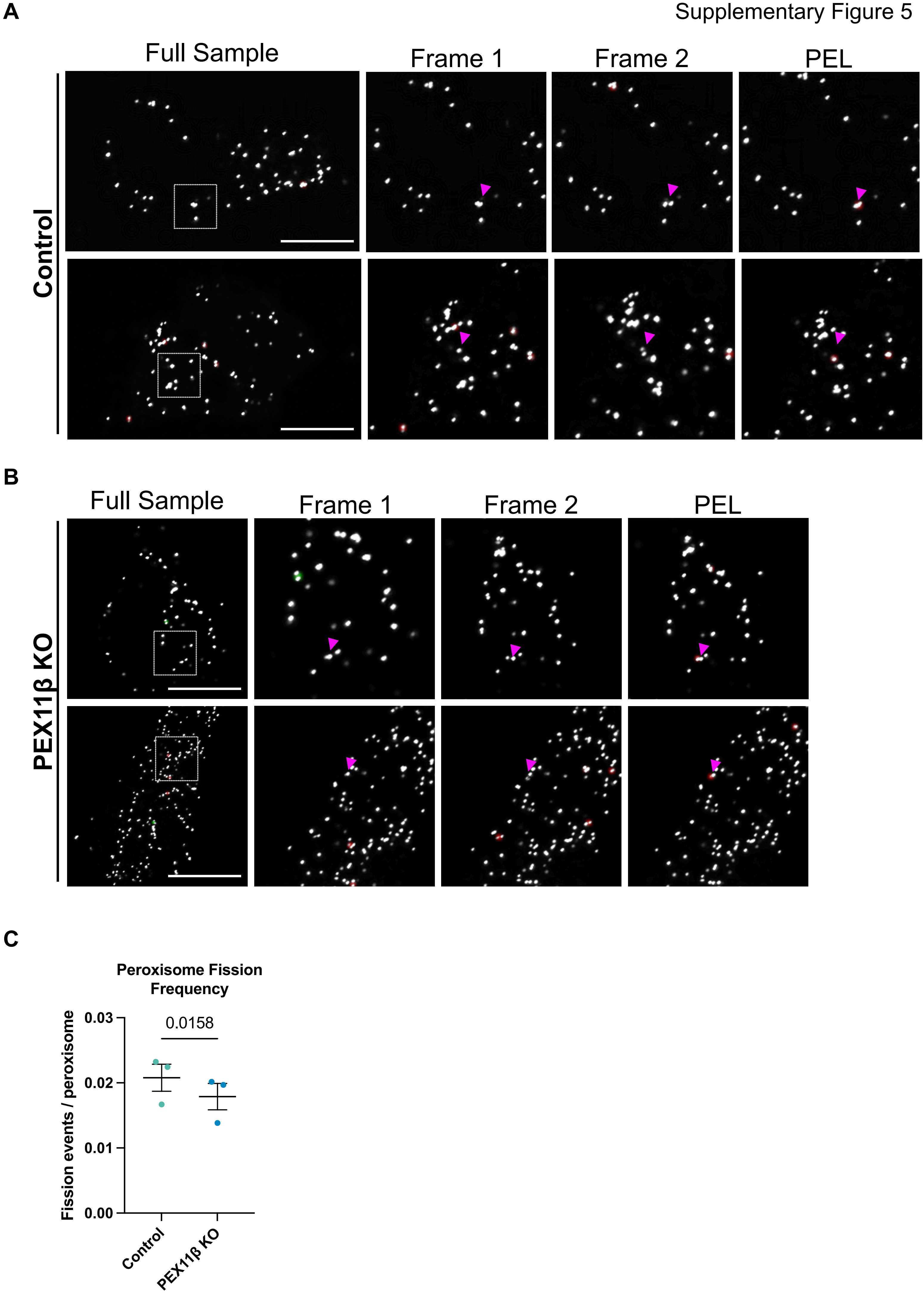

Supplementary Figure 6

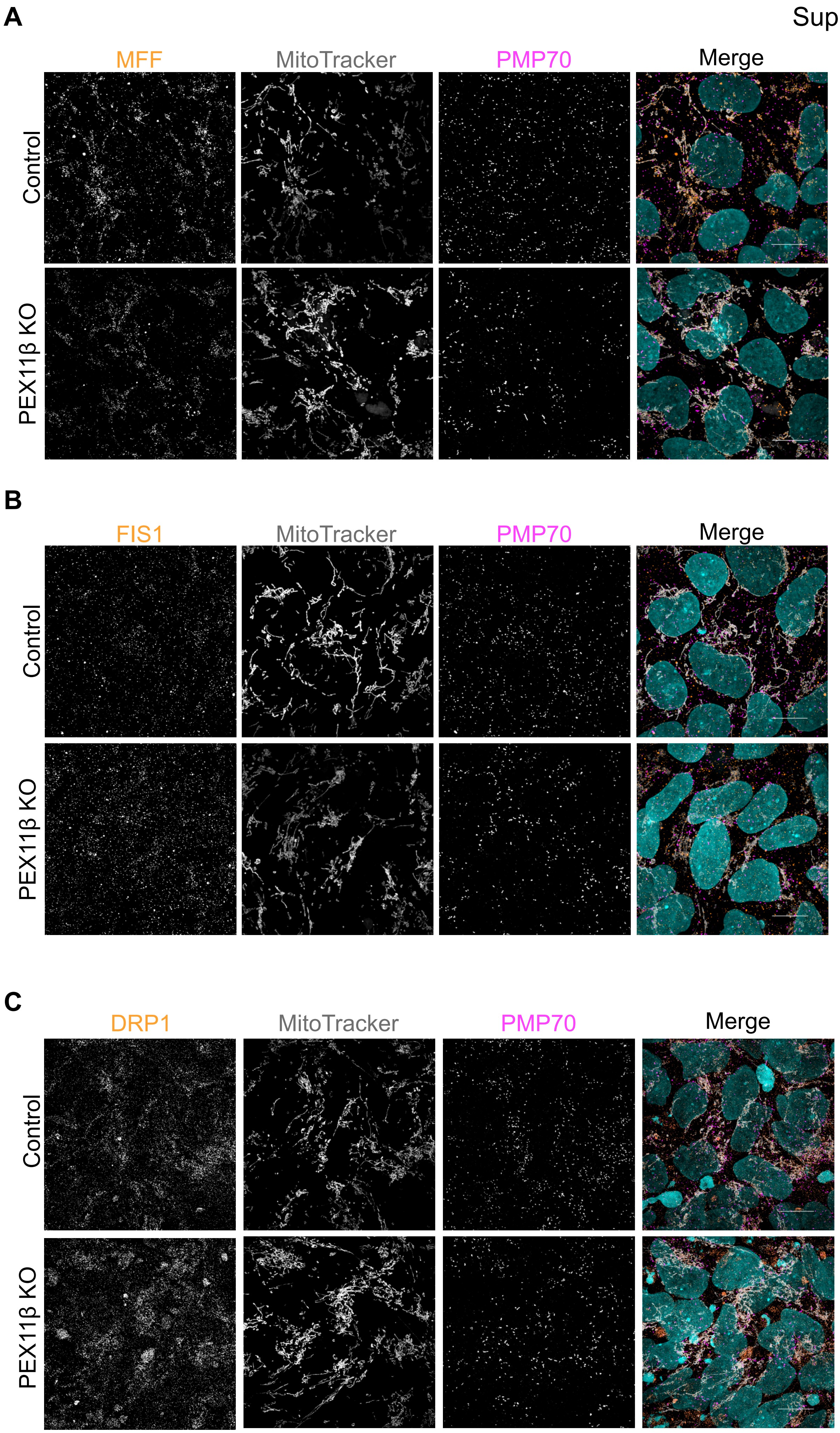

Supplementary Figure 7

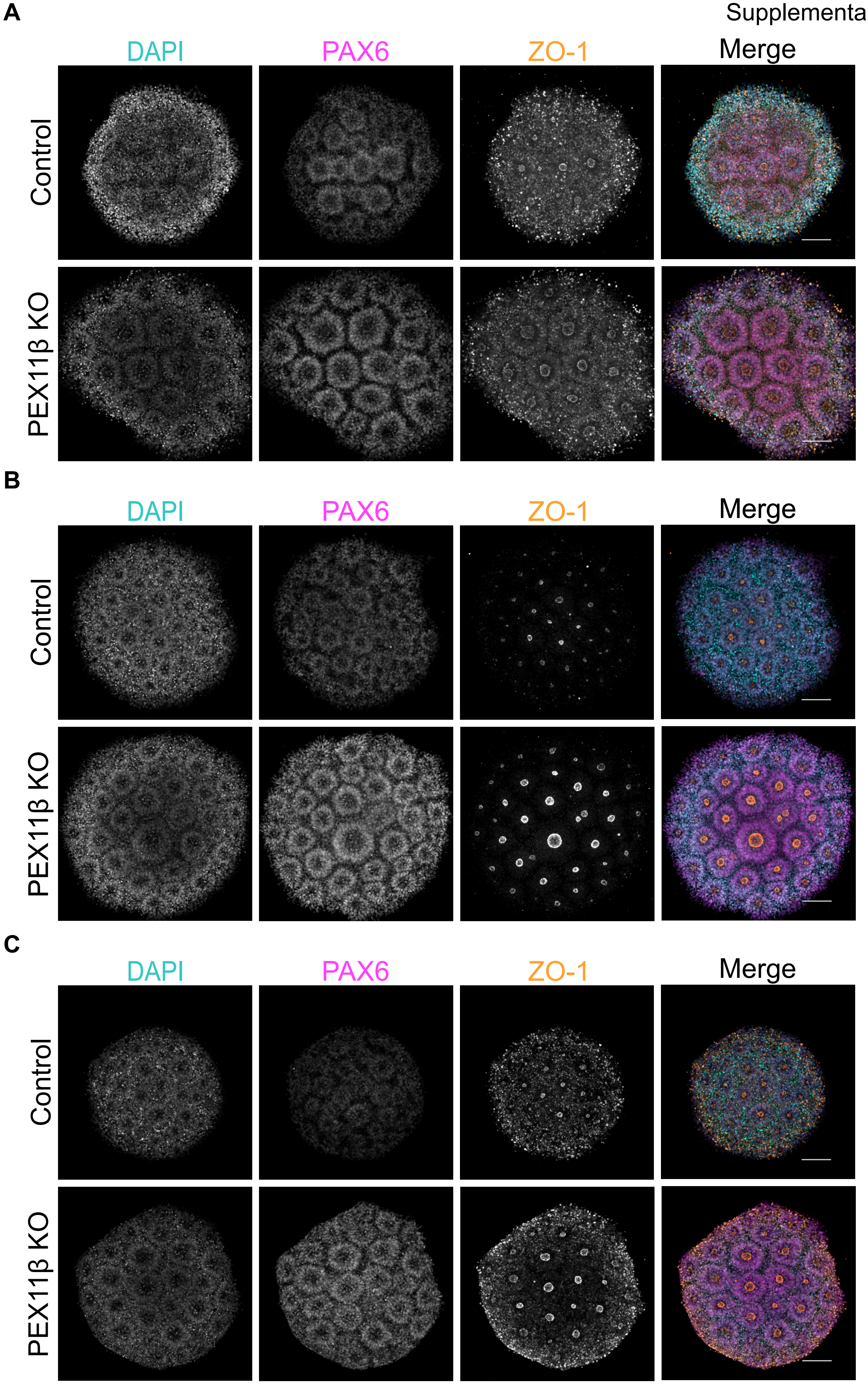

Supplement: Supplement 1 [file media-1.pdf]
